# Postoperative diabetes insipidus: how to define and grade this complication?

**DOI:** 10.1007/s11102-020-01083-7

**Published:** 2020-09-29

**Authors:** Friso de Vries, Daniel J. Lobatto, Marco J. T. Verstegen, Wouter R. van Furth, Alberto M. Pereira, Nienke R. Biermasz

**Affiliations:** 1 Department of Medicine, Division of Endocrinology, Leiden University Medical Center, Albinusdreef 2, Leiden, Postbox 9600, 2300 RC The Netherlands; 2 Department of Neurosurgery, Leiden University Medical Center, Albinusdreef 2, Postbox 9600, 2300 RC Leiden, The Netherlands; 3 Centre for Endocrine Tumors Leiden (CETL), Leiden University Medical Center, Albinusdreef 2, Postbox 9600, 2300 RC Leiden, The Netherlands

**Keywords:** Diabetes insipidus, Fluid imbalance, Complications, Transsphenoidal surgery, Pituitary tumor, Vasopressin

## Abstract

**Purpose:**

Although transient diabetes insipidus (DI) is the most common complication of pituitary surgery, there is no consensus on its definition. Polyuria is the most overt symptoms of DI, but can also reflect several physiological adaptive mechanisms in the postoperative phase. These may be difficult to distinguish from and might coincide with DI. The difficulty to distinguish DI from other causes of postoperative polyuria might explain the high variation in incidence rates. This limits interpretation of outcomes, in particular complication rates between centers, and may lead to unnecessary treatment. Aim of this review is to determine a pathophysiologically sound and practical definition of DI for uniform outcome evaluations and treatment recommendations.

**Methods:**

This study incorporates actual data and the experience of our center and combines this with a review of literature on pathophysiological mechanisms and definitions used in clinical studies reporting of postoperative DI.

**Results:**

The occurrence of excessive thirst and/or hyperosmolality or hypernatremia are the best indicators to discriminate between pathophysiological symptoms and signs of DI and other causes. Urine osmolality distinguishes DI from osmotic diuresis.

**Conclusions:**

To improve reliability and comparability we propose the following definition for postoperative DI: polyuria (urine production > 300 ml/hour for 3 h) accompanied by a urine specific gravity (USG) < 1.005, and at least one of the following symptoms: excessive thirst, serum osmolality > 300 mosmol/kg, or serum sodium > 145 mmol/L. To prevent unnecessary treatment with desmopressin, we present an algorithm for the diagnosis and treatment of postoperative DI.

## Introduction

Pituitary tumors require specialized care in particular for surgical management [26]. Centralization of care for patients with a pituitary tumor in Pituitary Centers of Excellence that deal with high surgical volumes and harbor multidisciplinary teams is advocated to minimize disease- and treatment-related morbidity [15]. Local audits within treatment facilities, but also comparison of treatment results and complications between centers, hold the promise for future quality assurance. Uniform application of the same definitions is mandatory to reliably measure and compare outcomes. However, establishing uniform definitions is challenging, especially for early postoperative complications such as diabetes insipidus (DI) and postoperative hyponatremia.

Postoperative DI is caused by vasopressin deficiency and is one of the most reported complications after pituitary tumor surgery [33]. While in the future measurement of copeptin might be a viable alternative to vasopressin measurement and diagnose DI, to date, diagnosis of DI is based on clinical and indirect laboratory findings as serum vasopressin measurement is expensive and results are not available within the time frame necessary in this setting. The majority of studies reporting on the incidence of postoperative DI do not provide uniform definitions. There is variation in the use of clinical and biochemical parameters which limits reliable comparisons between studies (Table 1) and results in reported incidence range between 2 and 54% [6, 18]. Although there is evidence on the etiology, prevalence numbers, variety of DI patterns, predisposing factors, and treatment of postoperative diabetes insipidus, a formal and widely used definition or consensus statement is not yet available [12, 21, 33].

**Table 1:** Overview of diagnostic criteria for DI as reported in literature

Category	Used criteria
Polyuria	UP > 250 mL/hr for 2 consecutive hours [22]
	UP > 300 mL/hr for 2 consecutive hours [33]
	UP > 300 mL/hr for 3 consecutive hours [2, 24, 32, 36]
	UP > 350 mL/hr for 2 consecutive hours [1]
	UP > 500 mL/hr for 3 consecutive hours [29]
	UP > 2500 mL/day [13, 17]
	UP > 4000 mL/day [10]
	UP > 3000 mL/day [25]
	UP > 5000 mL/day [40]
	UP relative to body weight [1, 10, 29, 32, 41]
Hypotonic urine	USG < 1.003 [31]
	USG < 1.005 [2, 10, 13, 18, 23, 24, 29, 34, 36, 40, 42]
	USG < 1.010 [1]
	Urine osmolality < 200 mosmol/kg [10, 23, 41]
	Urine osmolality < 300 mosmol/kg [1, 26, 29]
Serum osmolality	Na^+^ > 140 mmol/L [17]
	Na^+^ > 142 mmol/L [1]
	Na^+^ > 143 mmol/L [22] [26]
	Na^+^ > 145 mmol/L [2, 25, 29, 30, 32, 34, 41]
	Na^+^ > 148 mmol/L [42]
	Serum osmolality > 295 mosmol/kg [41]
	Serum osmolality > 300 mosmol/kg [1, 24, 33]
Other	Thirst [13, 18, 23, 28, 41]
	Hypovolemia [41]
	Exclusion of glycosuria [1, 18, 36, 40, 41]
	Polyuria resolves after desmopressin treatment [1, 28]

*UP* Urine production, *USG* Urine specific gravity, *Na*^+^ serum sodium

The primary objective of this review is to compose a definition and accompanying grading scheme for postoperative polyuria and DI. This study incorporates our data and experience from our center and a review of literature on pathophysiological mechanisms and definitions used in clinical studies reporting on postoperative DI.

### Physiological Control of Vasopressin Synthesis and Release

Vasopressin (also named arginine-vasopressin (AVP) or antidiuretic hormone (ADH)) is synthesized as a pre-prohormone (CT-proAVP) in the supraoptic and paraventricular nucleus of the hypothalamus and subsequently transported via axons through the pituitary stalk to the axon terminals in the posterior pituitary gland where it is stored in neurosecretory granules. In these granules, CT-proAVP is cleaved into vasopressin, neurophysin II, and copeptin. Upon physiological stimulation (increased plasma osmolality sensed by osmoreceptors) these peptides are released into the systemic circulation [9, 33, 41]. Vasopressin release is also stimulated during stress responses and as such co-stimulates adrenocorticotropic hormone (ACTH) secretion [31].

## Evaluation of the condition and proposal for grading of complication

### Postoperative fluid imbalances, differential diagnosis and contributing components

Fluid imbalances are highly prevalent after pituitary surgery (50–75.4% of cases) [14, 18] and are often a consequence of perioperative administration of high volumes of intravenous fluids and physiological adaptation of patients to a variety of stressors [18, 25]. The posterior pituitary gland plays a major role in fluid homeostasis which makes patients undergoing pituitary surgery more vulnerable to fluid imbalances due to insufficient or inappropriate vasopressin release. Polyuria following pituitary surgery can be the physiological response to fluid overload, but also the manifestation of osmotic diuresis or DI. The core symptoms of these causes of polyuria are summarized in Table 2.

**Table 2:** Diagnostic criteria for different states of fluid imbalances

	Fluid overload	Osmotic diuresis	Diabetes insipidus	Adipsic diabetes insipidus
Polyuria	Yes	Yes	Yes	Yes
USG	Normal or low	High	Low	Low
Thirst	Absent to low	Excessive	Excessive	Absent to low
Hyperosmolality/Hypernatremia	No	No	Absent to mild	Yes, can be severe

*USG* Urine Specific Gravity

#### Postoperative polyuria due to fluid overload

During pituitary surgery, the vast majority of patient receive large amounts of intravenous fluids. Based on sympathetic stimulation during surgery vasopressin and aldosterone secretion is stimulated, promoting fluid retention [18]. Moreover, patients often receive supraphysiologic dosages of glucocorticoids, which further promotes vasopressin release as well as water and salt retention via mineralocorticoid receptor stimulation in the kidneys. After surgery, vasopressin, aldosterone, and glucocorticoid concentrations decrease, resulting in the release of the retained fluids. This form of polyuria is transient in case of normal posterior pituitary function as adequate control of vasopressin release will normalize urinary output and restore fluid imbalances after excretion of the surplus of fluids.

A condition-specific form of postoperative polyuria occurs in patients with acromegaly and Cushing’s disease. In acromegaly, fluid and sodium retention occur as the result of the growth hormone (GH) and insulin-like growth factor I (IGF-I) excess. Consequently, abrogation of GH excess by successful adenectomy will result in a negative fluid balance during the first 48 h after surgery [17, 42]. In Cushing’s disease, cortisol excess facilitates fluid and sodium retention via overstimulation of mineralocorticoid receptors in the kidneys, whereas successful reduction of cortisol secretion will also result in increased fluid excretion [16].

#### Osmotic diuresis

Osmotic diuresis can occur as a result of glycosuria in patients with uncontrolled or undiagnosed diabetes mellitus or patients receiving high doses of corticosteroids. The prevalence of diabetes mellitus is higher both in acromegaly and Cushing’s disease patients due to the hormone excess [11, 22].

#### Diabetes insipidus

Central DI is a condition characterized by the inability to sufficiently concentrate urine due to impaired vasopressin release and can be caused by injury to the posterior pituitary gland, pituitary stalk or hypothalamus. DI will lead to extreme thirst, most typically for cold water, to compensate for the fluid loss. When fluid loss exceeds the patient’s ability to drink dehydration and hypernatremia may occur [21].

*Adipsic DI* can occur in patients with an impaired sense of thirst. Adipsic DI is extremely rare and is most likely caused by hypothalamic damage and can occur after extensive surgery in the hypothalamic region, which is more often seen in patients with large craniopharyngiomas and giant adenomas [8, 12, 37]. These patients do not feel an urge to compensate for the fluid loss by drinking water; adipsic DI renders the patient at risk of hypernatremia and severe dehydration.

Postoperative DI may be transient or permanent. *Transient DI* typically occurs within 24–48 h after surgery and resolves during the next couple of days. It is most likely caused by mild and reversible injury to the pituitary stalk or posterior pituitary lobe [18, 39]. *Permanent DI* occurs when the hypothalamus and/or pituitary stalk are irreversibly injured.

Postoperative DI can occur in combination with an episode of the Syndrome of Inappropriate ADH-secretion (SIADH). SIADH occurs as injured neurons of the hypothalamo-pituitary tract degenerate and release all stored vasopressin and most typically becomes manifest 5–8 days postoperative as it takes time for neurons to fully degenerate. SIADH renders patients receiving desmopressin treatment for preceding DI prone for hyponatremia [25, 33]. In the *biphasic pattern,* normal fluid balance is restored after the episode of SIADH (DI-SIADH-normal fluid homeostasis). In case no restoration to the posterior pituitary tract has occurred, the typical, but rare, *triphasic pattern* occurs and (permanent) DI will resume (DI-SIADH-DI) [14, 25, 33].

### Proposed definition for diagnosis of DI and rationale for this proposition

Mandatory criterium: polyuria (urinary output > 300 ml/h for 3 h) AND urine specific gravity (USG) < 1.005, in addition to at least one relative criterion: excessive thirst, serum osmolality > 300 mosmol/kg or serum sodium > 145 mmol/L (Box 1).

Proposed definition of postoperative Diabetes Insipidus. DI: Diabetes Insipidus, USG: Urine Specific Gravity, NRS: Numeric Rating Scale Hypotonic polyuria:

- Urine production > 300 mL/h for 3 consecutive hours
  AND
- USG < 1.005

And at least one of the following:

- Excessive thirst (NRS ≥ 6 out of 10)
- Serum osmolality > 300 mosmol/kg
- Serum sodium > 145 nmol/L.

#### Polyuria

DI typically has an abrupt onset and can lead to significant volume depletion in a short period, which warrants immediate and adequate surveillance. Therefore, the preferred criterion of polyuria is a urinary output of > 300 mL/h for more than 3 consecutive hours. With a time interval of 3 h, a timely increase of monitoring and intervention is possible. Correcting for bodyweight is useful in a pediatric setting as polyuria in children is usually defined as a urine production of > 2L/m^2^/24 h. A urine production of > 5–6 ml/kg/h is compatible with postoperative DI in children [27].

#### Low urine specific gravity

USG is widely used as a diagnostic tool in DI. It is easily applicable and does not require venipuncture. Normal USG ranges from 1010 to 1030, whereas osmotic diuresis results in a higher USG and DI in diluted urine with very low USG. Therefore, we propose to use the commonly used cut-off point of USG < 1005.

#### Thirst

Unquenchable thirst will precede hyperosmolality and hypernatremia in most cases as it is an early sign of increasing plasma osmolality and a prerequisite for restoring osmolality. This enables clinicians to use thirst as an early diagnostic criterion for DI [18]. Attention should be given to distinguish thirst due to a dry mouth as a result of nasal packing and unquenchable thirst as a result of volume depletion in DI. The former will resolve by watering the mouth with small sips, whereas the latter will not. Unquenchable thirst can be the only relative criterion when a patient is able to maintain a neutral fluid balance with increased fluid intake. It is important to note that the physiological trigger of thirst cannot be used as a criterion in the situation of adipsic DI or altered neurological state. Although thirst is a subjective measure, abnormal thirst is easier to assess for patients whereas abnormal excessive fluid intake may go unnoticed. This is mainly due to the wide variation of normal fluid intake between individuals without them knowing their usual intake, which might delay diagnosing DI. To quantify thirst, a numerical rating scale (NRS) has been used in previous studies, which correlated highly with plasma osmolality during a water deprivation test [3].

#### Plasma osmolality, serum sodium

As soon as an absolute water deficit occurs, hyperosmolality and hypernatremia are strong indicators of DI. The most reliable measure of a patients’ intrinsic fluid balance is serum osmolality. However, in patients without uncontrolled diabetes mellitus or kidney failure, serum sodium is the most important denominator of serum osmolality. In uncontrolled diabetes mellitus, dilutional hyponatremia may occur as a result of high plasma glucose levels. As serum sodium is a simple and readily available measure it can be used as a substitute for serum osmolality if uncontrolled diabetes mellitus or kidney failure can be excluded.

### Proposed grading of postoperative diabetes insipidus as a complication

*Grade 0: Unlikely DI,* probably physiological or osmotic polyuria (no complication).

*Grade 1: Probable Transient* DI, spontaneously resolving within 48 h after surgery, which does not prolong hospital stay, regardless of incidental desmopressin administration (no complication).

*Grade 2: Transient* DI, which requires treatment for < 2 weeks after surgery and may prolong length of hospital stay (Clavien-Dindo class II).

Grade 3: *Prolonged* DI, which requires treatment for a minimum of 2 weeks, but fewer than 6 months (Clavien-Dindo class II).

*Grade 4: Chronic (persisting)* DI which requires treatment for more than 6 months (Clavien-Dindo class II) (Box 2).

The cut-off of 2 weeks is based on the typical resolution of transient DI in this time frame. The cut-off of 6 months is used as patients with DI over 6 months rarely restore normal fluid homeostasis. As restoration does occur sometimes, we encourage endocrinologists to occasionally evaluate necessity of desmopressin use during long-term follow-up by asking if the patient notices increased urinary output when administration of the next dose is due or if they forget a dose. Moreover, as this grading is to be used in scientific reporting, it is also based on the fact that surgical cohorts often report their outcomes at 6 months postoperative.

Proposed grading of postoperative Diabetes Insipidus. DI: Diabetes Insipidus Grade:

1. *Probable Transient* DI: spontaneously resolving within 48 h after surgery and no prolongation of hospital stay, no clear need for desmopressin

Diabetes insipidus necessitating treatment:

2. *Transient* DI, < 2 weeks
3. *Prolonged* DI, > 2 weeks, but < 6 months
4. *Chronic (persisting)* DI: > 6 months

### Occurrence of DI in the leiden cohort (Table 3)

**Table 3:** Prevalence of DI and SIADH in the Leiden cohort of 474 endoscopic transsphenoidal surgeries for pituitary tumors

Type of imbalance	No. (%)
DI	120 (25.3)
Grade 1	25 (5.3)
Grade 2	34 (7.2)
Grade 3	32 (6.8)
Grade 4	29 (6.1)
SIADH	55 (11.6)
Isolated DI	106 (22.4)
Isolated SIADH	41 (8.6)
Biphasic pattern	10 (2.1)
Triphasic pattern	4 (0.8)

*DI* Diabetes Insipidus, *SIADH* Syndrome of Inappropriate ADH-secretion

As an example, we analyzed all 474 patients that underwent endoscopic pituitary surgeries at our specialized pituitary clinic, which is a member of Endo-ERN (www.endo-ern.eu). This cohort was previously described [20]. A form of DI occurred in 25% of cases (n = 120). Following the aforementioned criteria, transient DI occurred in 91 cases (19.2%) (Grade 1: n = 25 (5.3%), Grade 2: n = 34 (7.2%), grade 3: n = 32 (6.8%)) and chronic (grade 4) DI in 29 cases (6.1%).

## Management of DI

In this section, we present the algorithm for diagnosis and treatment of postoperative DI that was reached consensus on at a local level to aid centers that do not have an operational protocol or are yet in the process of developing one. We would like to stress that the diagnostic and treatment process is complex and is both patient and situation dependent. Other centers might use different or adjusted protocols to satisfaction and with similar treatment results. Ideally, a consensus meeting between expert centers or within networks (as Endo-ERN) would be planned to take this further towards a general guideline for postoperative DI.

### Diagnosis of postoperative DI (Fig. 1)

**Fig. 1:**
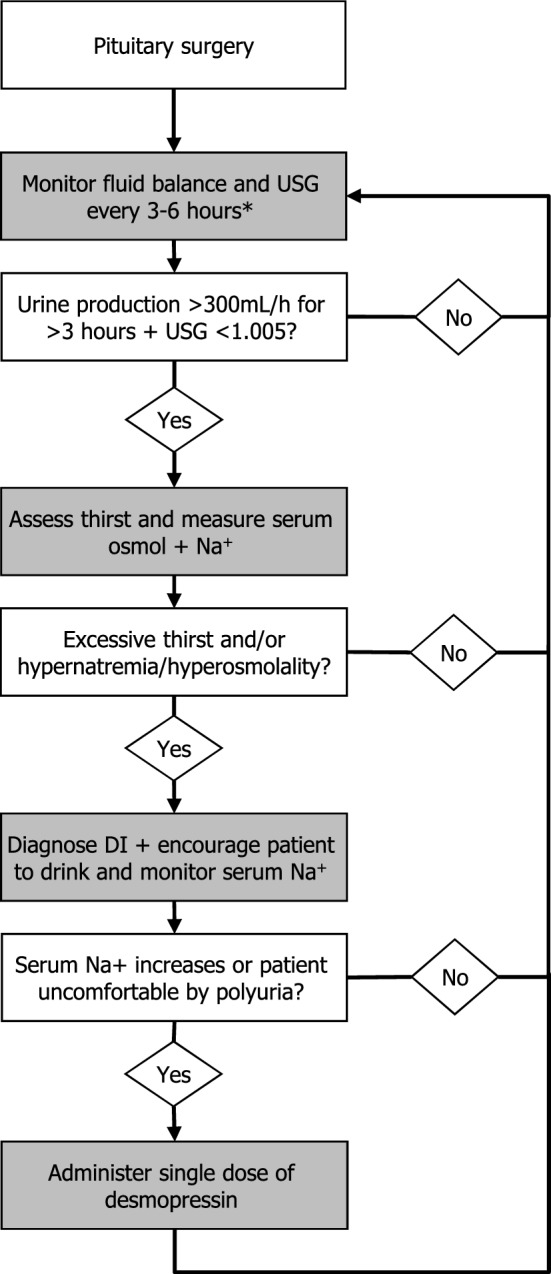
Flow diagram for the proposed diagnosis and initial treatment of postoperative Diabetes Insipidus during first 3 days postoperative *3 h for high-risk patients (hypothalamic involvement (adipsic diabetes insipidus) or diagnosed diabetes insipidus), 6 h for other patients. *USG* Urine Specific Gravity, osmol: osmolality, *Na*^+^ serum sodium, *DI* Diabetes Insipidus

Fluid balance should continuously be monitored at six-hour intervals after surgery. A three-hour interval should be used in patients at high risk for DI or experiencing a current episode of DI (see Fig. 1). Specific attention should be given to the fluid balance during transfers between locations (e.g. from the operation room to the recovery room, recovery room to ward, intensive care unit to ward). Furthermore, unnecessary perioperative fluid administration of in the holding or operation room should be discouraged. As glucocorticoids suppress vasopressin release DI in patients with new adrenal insufficiency without glucocorticoid treatment may not become manifest and underestimated. Specific attention should be given to patients in which glucocorticoid treatment is initiated as an episode of DI may be “unmasked” [4].

These recommendations have led to the following assessment scheme for polyuria (> 300 mL for > 3 consecutive hours), which follows a step-wise approach:

1. Assessment of urine specific gravity in each urine portion (see Table 1)
2. If USG is < 1005, assess the presence of unquenchable thirst, preferably with the NRS and measure serum osmolality and/or serum sodium concentration.
3. In case of unquenchable thirst and/or an increased serum sodium concentration (> 145 mmol/L): diagnose postoperative DI, and treat accordingly (see next paragraph).
4. Repeat the assessment as stated above every 3 h until the urine production normalizes.

### Treatment of postoperative DI

Step 1: (Mild) Postoperative DI can initially be treated with adequate water intake; compensating the excretion with equivalent intake. Patients should be encouraged to drink according to thirst to compensate for the fluid loss and prevent hypernatremia.

Step 2: The fluid balance and electrolyte status should be monitored every 3 h to assess if the fluid loss is adequately compensated.

Step 3: When the fluid loss exceeds the patient’s ability to drink or causes discomfort, incidental desmopressin, a synthetic vasopressin-analogue, should be administered. In our center, we administer desmopressin orally in 50 to 100 µg doses, but nasal, subcutaneous, and intravenous preparations are also available. However, nasal administration is not appropriate in the postoperative period due to nasal congestion causing absorption impairment. After administration of desmopressin, the fluid balance and electrolyte status of the patient should be monitored every 3 h. Attention should be given to fluctuating severity and the possibility of the development of SIADH.

### Patients at risk for adipsic diabetes insipidus

In cases with surgery and hypothalamic involvement and neurological symptoms, there should be additional awareness of the likelihood of adipsic DI as acute changes in osmolality and severe hypernatremia can cause potentially life-threatening situations. In such patients, serum osmolality or serum sodium should be analyzed proactively and frequently (e.g. every 6 h). An additional tool to assess volume depletion in these patients is daily weight measurement. The threshold for administration of desmopressin or iv fluids should be low.

### Risk factors for postoperative diabetes insipidus

A limited number of studies reported on an association between preoperative clinical factors and the occurrence of postoperative DI, but a recent systematic review on risk factors for postoperative complications found incongruous results [19]. Some studies describe a higher incidence in Rathke’s cleft cysts (RCC) [35, 36], or larger tumors [7, 23], whereas other studies do not confirm these findings [35, 36, 38].

## Future perspectives: copeptin

Recent studies have analyzed the possibility of using copeptin as a marker for postoperative DI. The benefit of copeptin over vasopressin is that it is more stable and can be measured more reliably. Winzeler et al. found that a low serum copeptin after surgery was a prognostic factor for DI, especially when blood samples were drawn within 12 h post-surgery [41]. Additionally, Berton et al. demonstrated that the absence of a copeptin peak one hour after extubation was a reliable predictor of postoperative DI [5]. If a clinically useful cut-off for copeptin with very high sensitivity can be established, patients with copeptin levels exceeding this cut-off do not need to be subjected to close monitoring of the fluid balance for the possible occurrence of DI.

## Conclusion

Postoperative DI is a common complication after pituitary surgery. However, it can be very difficult to distinguish DI from other types of polyuria. DI can manifest in different ways: transient, permanent and with/without an episode of SIADH. We propose a pathophysiological and literature-based definition and treatment regimen. This will increase clarity and uniformity for physicians and will increase comparability between studies in an attempt to further ameliorate post-surgical care for patients undergoing pituitary surgery. We regard this manuscript as a prelude for future discussions between reference centers to reach consensus in how to prevent and treat postoperative DI and how to reach uniformity in definition, grading and reporting of this complication.

## Data Availability

Data will be stored for 15 years after publication.
